# Hypoxia Inducible Factor as a Central Regulator of Metabolism – Implications for the Development of Obesity

**DOI:** 10.3389/fnins.2018.00813

**Published:** 2018-11-01

**Authors:** Joana M. Gaspar, Lício A. Velloso

**Affiliations:** ^1^Post-Graduation in Biochemistry, Department of Biochemistry, Federal University of Santa Catarina, Florianópolis, Brazil; ^2^Laboratório de Bioenergética e Estresse Oxidativo, Departamento de Bioquímica, Centro de Ciências Biológicas, Universidade Federal de Santa Catarina, Florianópolis, Brazil; ^3^Laboratory of Cell Signaling, Obesity and Comorbidities Research Center, University of Campinas, Campinas, Brazil; ^4^National Institute of Science and Technology on Neuroimmunomodulation, Rio de Janeiro, Brazil

**Keywords:** HIF complex, obesity, metabolic disorders, inflammation, hypothalamus, energy homeostasis

## Abstract

The hypothalamus plays a major role in the regulation of food intake and energy expenditure. In the last decade, it was demonstrated that consumption of high-fat diets triggers the activation of an inflammatory process in the hypothalamus, inducing neurofunctional alterations and contributing to the development of obesity. Hypoxia-inducible factors (HIFs) are key molecules that regulate cellular responses to inflammation and hypoxia, being essential for the normal cell function and survival. Currently, evidence points to a role of HIF pathway in metabolic regulation that could also be involved in the progression of obesity and metabolic diseases. The challenge is to understand how HIF modulation impacts body mass gain and metabolic disorders such as insulin resistance. Distinct animal models with tissue-specific knocking-out or overexpression of hypoxia signaling pathway genes revealed a cell-specificity in the activation of HIF pathways, and some of them have opposite phenotypes among the various HIFs gain- and loss-of-function mouse models. In this review, we discuss the major findings that provide support for a role of HIF pathway involvement in the regulation of metabolism, especially in glucose and energy homeostasis.

## Introduction

Obesity has reached epidemic proportions around the world. It is characterized by excessive and abnormal fat mass accumulation, associated with chronic low-grade systemic inflammation, which predispose to metabolic diseases. In 2015, in the adult population, a total of 603.7 million were obese and approximately 1.9 billion were overweight. In addition, childhood obesity has increased considerably raising concern issues on the future of public health in the world (Collaborators et al., 2017; Gregg and Shaw, 2017).

Obesity has a multifactorial and complex etiology, involving the combination of genetic and epigenetic factors, life style, and socioeconomic status, which result in increased food intake, decreased energy expenditure, and also changes in nutrient utilization and metabolism, ultimately leading to a positive energy balance. The aberrant expansion of white adipose tissue, through hyperplasia as well as hypertrophy, promotes insufficient intra-adipose blood perfusion resulting in hypoxia. These niches of hypoxia are partially responsible for white adipose tissue inflammation and insulin resistance (Jiang et al., 2011; Lee et al., 2011, 2014; Krishnan et al., 2012; Michailidou et al., 2015). Hypoxia-inducible factor-α (HIF-α) is a fundamental transcription factor involved in the oxygen homeostasis that induces inflammation and insulin resistance in obesity. In addition, the abnormal regulation of HIF complex is involved in the development of obesity, fatty liver disease, and type 2 diabetes (Virtue and Vidal-Puig, 2011; Girgis et al., 2012). Moreover, recent data show that HIF has important functions in the hypothalamus, the region of the brain that controls energy balance and metabolism (Lando et al., 2002; Bento and Pereira, 2011; Zhang et al., 2011; Ban et al., 2014).

Currently, the approaches used to treat obesity include behavioral counseling for reducing caloric intake and increasing physical activity, the use of some pharmacological compounds that reduce hunger and caloric harvesting from the gut and bariatric surgery. However, these interventions have not proven effective in providing long term weight loss, highlighting the importance of development of new strategies to tackle obesity (Gonzalez-Muniesa et al., 2017; Narayanaswami and Dwoskin, 2017; Srivastava and Apovian, 2018). Several animal studies manipulating HIF pathways highlight the involvement of this transcriptional factor in the pathogenesis of obesity placing it as a potential target for the treatment of obesity and diabetes. The current challenge is to understand how HIF modulation might impact body weight gain and metabolic disorders such as insulin resistance. Here, we review data that have contributed to expand the current understanding of how HIF regulates whole body metabolism and how it is affected by nutritional and immune signals that are involved in the pathophysiology of obesity and metabolic disorders.

## Hypothalamic Regulation of Energy Expenditure

The regulation of energy homeostasis is a highly integrated and regulated process aimed at maintaining the stability of body energy stores over time. Neurons located in the hypothalamus are the most important components of the complex system that regulates energy flux in the body. Hypothalamic neurons respond to peripheral signals controlling food intake and energy expenditure on a homeostatic way, and also integrate with limbic and cortical areas to coordinate the hedonic responses to food (Timper and Bruning, 2017; Rossi and Stuber, 2018).

The hypothalamus, located at the base of the forebrain and around the walls of third ventricle, is at a privileged location to receive afferent signals from the periphery through the bloodstream, and also from the brainstem. These signals inform hypothalamic neurons about the magnitude of the energy stores in body. In turn, the neurons respond providing signals that regulate hunger and energy expenditure, as well as systemic glucose and lipid metabolism (Williams and Schwartz, 2011; Kim et al., 2018). The hypothalamus is composed by several nuclei arrayed in a three-dimensional organization, each with distinct neuronal connections and functions, including the arcuate nucleus (ARC), paraventricular nucleus (PVN), ventromedial nucleus (VMN), dorsomedial nucleus (DMN), and lateral hypothalamic area (LHA; Baroncini et al., 2012; Kim et al., 2018).

The role of the hypothalamus in feeding and metabolism was first demonstrated in the 1940s, when severe hypophagia was observed after large lesions centered on the lateral hypothalamus (Hetherington and Ranson, 1940). Conversely, lesions on the VMH and ARC induced hyperphagia and obesity (Marshall and Mayer, 1956; Olney, 1969). Such studies led to the definition of the central role played by the hypothalamic nuclei controlling satiety and hunger (Timper and Bruning, 2017; Kim et al., 2018).

The strategic location of ARC in proximity to the median eminence places its neurons in close contact with a special type of blood/spinal fluid interface that is less restrictive than the classical blood/brain barrier (Haddad-Tovolli et al., 2017). Due to this anatomical particularity, ARC neurons are exposed to systemic oscillations of hormone and nutrient levels providing rapid and dynamic information required for optimal regulation of whole body energy homeostasis. Within the ARC, there are three main neuronal subpopulations: a subpopulation of anorexigenic neurons that co-express cocaine and amphetamine-related transcript (CART) and proopiomelanocortin (POMC; Vrang et al., 1999; Yaswen et al., 1999); and two subpopulations of orexigenic neurons, one co-expressing neuropeptide Y (NPY) and agouti-related protein (AgRP; Hahn et al., 1998; Luquet et al., 2005), and another expressing tyrosine hydroxylase (TH; Zhang and van den Pol, 2015, 2016). ARC neurons communicate with other hypothalamic areas (PVN, DMN, and LHA) involved in appetite regulation.

### Hormonal Regulation

**FIGURE 1:**
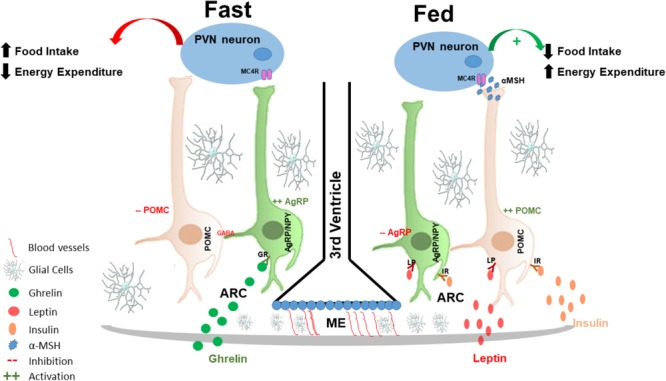
Hypothalamic regulation of energy homeostasis: the hypothalamus senses and integrates peripheral adipostatic hormones that circulate in levels proportionate to nutritional status and adipose tissue stores. Under fasting conditions ghrelin, secreted by the stomach, binds to its receptor in AgRP neurons in the ARC, leading to its activation. AgRP neuronal activation increased the release of AgRP that is an antagonist of MC4R in the PVN neurons; at the same time, AgRP neurons also release of GABA neurotransmitter that inhibited POMC neurons, and consequently increasing appetite and decreased energy expenditures. In the fed state, insulin and leptin act directly on their receptors, both in POMC and AgRP neurons in the ARC of the hypothalamus to control energy homeostasis; insulin and leptin induce inhibition of AgRP and activation of POMC neurons. POMC neuronal activation involves the processing of POMC with formation of α-MSH that is an agonist of MC4R and consequently activating PVN neurons. PVN activation culminates in satiety (decreased food intake) and stimulation of energy expenditure. AgRP, agouti related protein; MC4R, melanocortin 4 receptor; GABA, gamma-aminobutyric acid; POMC, proopiomelanocortin; α-MSH, alpha-melanocyte-stimulating hormone; PVN, paraventricular nucleus.

The identification of leptin in 1994 (Zhang et al., 1994) is a hallmark that provided advance in the characterization of hypothalamic pathways/mechanisms involved in the regulation of energy homeostasis. Leptin is secreted by adipose tissue and circulates at concentrations proportional to fat mass providing key signals for the regulation of food intake (Figure 1). Leptin binds to the long form of the leptin receptor, Ob-Rb, both in POMC and AgRP neurons in the hypothalamus. POMC neurons are activated by leptin, while AgRP neurons are inhibited (Cheung et al., 1997; Williams and Schwartz, 2011; Lee et al., 2018). Insulin is also an important regulator of food intake and energy expenditure. Insulin receptors are distributed all over the brain, particularly in hypothalamic neurons and upon ligand-induced stimulation it activates POMC neurons and inhibit food intake (Woods et al., 1979; Belgardt et al., 2008; Figure 1).

Activation of POMC neurons induces the release of α-melanocyte-stimulating hormone (α-MSH) that is an endogenous agonist of the melanocortin 3 and 4 receptors (MC3-R and MC4-R), mostly localized in the PVN (Cowley et al., 2001; Mountjoy, 2015). Conversely, AgRP is an endogenous antagonist of MC4-R in the PVN. The inhibition of AgRP neurons by leptin blocks the activation of PVN neurons, which results in the inhibition of synaptic activation, increasing food intake and consequently decreasing energy expenditure (Ollmann et al., 1997; Mizuno and Mobbs, 1999; Lee et al., 2018).

Ghrelin is yet another hormone that is involved in hypothalamic neuronal regulation. It is primarily produced by the stomach in fasting condition and has the property of inducing food intake, through the activation of ghrelin receptors expressed predominantly in AgRP neurons (Al Massadi et al., 2017). Thus, AgRP/NPY neurons are activated by fasting and ghrelin (Kamegai et al., 2000; Nakazato et al., 2001; Tang-Christensen et al., 2004; Betley et al., 2015), leading to a greater release of AgRP; therefore, stimulating appetite. Furthermore, AgRP neuronal activation also leads to the release of gamma-aminobutyric acid (GABA) and NPY that inhibit POMC neurons (Cowley et al., 2001; Atasoy et al., 2012) providing further increase in food intake.

### Nutrient Sensing

There are extensive data indicating that in addition to hormonal factors, hypothalamic neurons can also sense nutrients (glucose, lipids, and some amino acids, such as leucine) to modulate food intake and energy expenditure (Loftus et al., 2000) and also to regulate systemic glucose and lipid levels (Obici et al., 2002b; Chari et al., 2010; Thorens, 2012).

The first demonstration that hypothalamus can sense nutritional status came from studies of Miselis and Epstein (1975), showing that the induction of glucoprivation in hypothalamus increases feeding behavior. Thereafter, two hypothalamic neuronal subpopulations were defined as glucose-excited (GE), found mostly in the lateral ARC, and glucose-inhibited (GI) neurons, found in the medial ARC (Wang et al., 2004; Thorens, 2012). Later on, studies demonstrated that GE are predominantly POMC neurons (Parton et al., 2007), whereas GI are predominantly AgRP (Chalmers et al., 2014). Hypothalamic increase of glucose concentration results in a decrease in AgRP mRNA levels and to an activation of POMC neurons, which leads to decreased food intake, decreased hepatic glucose production and increased insulin secretion (Thorens, 2012).

Energy depletion is sensed by mechanisms that activate catabolic pathways to mobilize cellular components for the provision of energy, such as autophagy and 5′ AMP-activated protein kinase (AMPK). AMPK is a central enzyme for the regulation of whole body metabolism, its activation increases fatty acid oxidation, glucose uptake, and stimulates glycolysis and mitochondrial biogenesis (Ha et al., 2015). In POMC and AgRP neuronal populations, the activation of AMPK acts as a nutrient sensor and as a regulator of satiety (Claret et al., 2007; Kola, 2008), by altering the expression of orexigenic or anorexigenic neuropeptides. Alterations in hypothalamic glucose concentration act in part by modulating the activity of AMPK which is an important nutrient sensor in several tissues in the body, including the hypothalamus (Minokoshi et al., 2004; Claret et al., 2007; Lockie et al., 2018). Intracerebroventricular (icv) glucose infusion decreases AMPK activity in POMC neurons and consequently reduces food intake (Zhang et al., 2011). However, low glucose concentration induces activation of AMPK (in AgRP neurons) leading to increased food intake and body mass gain, this effect is inhibited by insulin and leptin. Briefly, AMPK is inhibited by high glucose concentrations or by refeeding that decrease the expression of AgRP and NPY (Claret et al., 2007; Chalmers et al., 2014). High glucose concentration can induce depolarization in POMC neurons leading to its activation (Parton et al., 2007). Thus, the combined actions of glucose in AgRP and POMC neurons provide anorexigenic signals through a pathway distinct but yet integrated with the pathway controlled by hormones.

Autophagy is an evolutionary conserved mechanism that provides an alternative form of energy to the starved cell. Some evidence suggests that autophagy is closely involved in the hypothalamic regulation of food intake (Kaushik et al., 2011, 2012; Singh and Cuervo, 2011; Portovedo et al., 2015; Oh et al., 2016). Long-term fasting was shown increase autophagy in AgRP neurons resulting in increased expression of the neuropeptide AgRP and thus, increasing food intake (Kaushik et al., 2011). In POMC neurons, the impairment of autophagy induces an increase in food consumption and body weight gain (Malhotra et al., 2015). Recently, it was demonstrated that autophagy that occurs in hypothalamic neuronal populations is induced/regulated by the activation of AMPK. Under low-glucose availability AMPK-induced autophagy regulates appetite by regulating neuropeptide expression NPY and POMC (Oh et al., 2016).

Fatty acids and the amino acid leucine also regulate food intake via hypothalamus. Enzymes and intermediates of fatty acid metabolism function as energy sensors for hypothalamic response to fats. The icv administration of long-chain fatty acids can trigger a hypothalamic response to inhibit food intake and regulate glucose homeostasis (decrease in plasma insulin and glucose levels and also reduction of hepatic glucose production; Obici et al., 2002a). The mechanism behind regulation of food intake is likely mediated by the decreased expression of orexigenic hypothalamic NPY and AgRP levels (Obici et al., 2002a; Morgan et al., 2004; Chari et al., 2010). A summarized view of the physiological hypothalamic control of food intake and energy expenditure is shown in Figure 1.

## Obesity-Associated Hypothalamic Dysfunction

In non-pathological conditions, hypothalamic neurons are programed to respond to hormones, nutrients, and neural signals maintaining whole body energy homeostasis. However, several studies have shown that during the development of obesity, a defective regulation of this system plays a major role in the emergence of a positive energy balance. The main mechanism behind abnormal hypothalamic function in obesity is a low-grade hypothalamic inflammation that is triggered by the increased consumption of dietary fats (De Souza et al., 2005; Cavadas et al., 2016; Guillemot-Legris and Muccioli, 2017; Jais and Bruning, 2017). Activation of Toll-like receptor-4 (TLR4), induction of endoplasmic reticulum stress, and activation of isozyme protein kinase C-theta (PKC-theta) have all been identified as important mediators of the dietary fats induced inflammation (Benoit et al., 2009; Milanski et al., 2009). The consumption of large portions of predominantly saturated dietary fats promotes a rapid (as early as one day) increase in the expression of proinflammatory cytokines in the hypothalamus. Most of this response is dependent on the activation of resident microglia (Thaler et al., 2012; Morari et al., 2014; Valdearcos et al., 2014, 2017). If the consumption of dietary fats is interrupted after short time, the inflammation will disappear without major damage. However, if the consumption of dietary fats persists, peripheral myeloid cells will be recruited to the hypothalamus and the chronicity of inflammation will provoke damage to neurons (Moraes et al., 2009; Souza et al., 2016; Valdearcos et al., 2017). Studies have shown that in diet-induced obesity, the hypothalamic neurons are afflicted by abnormal mitochondrial turnover, abnormal function of the ubiquitin/proteasome system, and abnormal regulation of autophagy (Horvath et al., 2010; Ignacio-Souza et al., 2014; Portovedo et al., 2015; Carraro et al., 2017). Initially, neurons become resistant to the actions of leptin and insulin; however, as inflammation persists, neuronal apoptosis will appear. POMC neurons are more sensitive to the damage inflicted by inflammation and after some time, an imbalance in the orexigenic and anorexigenic hypothalamic neuronal subpopulations can act to boost body mass gain (Souza et al., 2016). Taken together all of these studies suggest that hypothalamic neuroinflammation could be a cause rather than a consequence of diet-induced obesity and metabolic dysfunction. Defining the details involved in the regulation and response to hypothalamic inflammation may help identifying potential targets to prevent and treat obesity.

## HIF Canonical Pathway and Cellular Functions

Hypoxia-inducible factors (HIFs) are basic helix-loop-helix transcription factors that act as master regulators of hypoxia activated gene expression, allowing the adaptation to hypoxia (Semenza et al., 1991; Wang et al., 1995). HIF is a heterodimer complex composed by two subunits, α-subunit (oxygen sensitive) and a β-subunit [constitutively expressed, and also called aryl hydrocarbon receptor nuclear translocator (ARNT)]. In mammalian cells, there are three major α subunits isoforms, 1α, 2α (also known as endothelial PAS domain-containing protein 1, EPAS1), and 3α. HIF-1α subunit is ubiquitously expressed and 2α is selectively expressed in tissue-restricted manner (e.g., in vascular endothelial cells and myeloid-derived cells). Both HIF1α and HIF2α function mainly as transcriptional activators, regulating several biological processes, such as angiogenesis, glucose, fatty acid, cholesterol, and mitochondrial metabolism and inflammation (Greer et al., 2012; Semenza, 2014). HIF3α has very weak transcriptional capacity, compared to other HIFs isoforms. A splicing isoform generated from HIF3α, inhibitory PAS (Per/Arnt/Sim) domain protein (IPAS), can function as a dominant negative regulator of HIF1α. This splicing isoform of HIF3 α can function as a negative feedback loop regulation of adaptive responses to hypoxia/ischemia (Makino et al., 2002).

**FIGURE 2:**
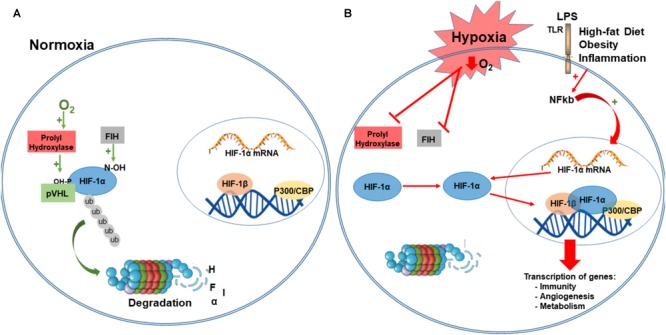
Hypoxia-inducible factor-1 regulation. Under normoxia, HIF-1α is hydroxylated on proline residues 402 and 564 by prolyl hydroxylases (PHD). This hydroxylation is a target for von Hippel-Lindau protein (VHL) binding, a E3 ligase that targets HIF-1α for ubiquitination and proteasomal degradation. Factor inhibiting HIF-1 (FIH-1) hydroxylated an asparagine residue (803) that blocks the binding of the coactivator p300/CBP **(A)**. Under hypoxia, PDH and FIH are inhibited and HIF-1α is stabilized to dimerize with HIF-1β, and binds to target genes at the DNA, recruits p300, and activates transcription of several genes involved in immunity, angiogenesis, metabolism, survival, and proliferation. Immune and inflammatory stimuli can also regulate the HIF pathway, primarily via activation of Toll-like receptors (TRL) and NF-kB activation. NF-Kb is translocated to the nucleus where it regulates the expression of HIF- α mRNA **(B)**. CBP/p300, Creb Binding Protein/p300; LPS, lipopolysaccharide; PHD, prolyl hydroxylases; VHL, von Hippel-Lindau protein; FIH-1, factor inhibiting HIF-1; P-OH, hydroxylation in proline residues; N-OH, hydroxylation in asparaginyl residues; TLR-4, Toll-like receptors; ub, ubiquitin; NF-kB, nuclear factor kappa-light-chain-enhancer of activated B cells.

Under normoxia, the hydroxylation of a proline and asparagine residues suppresses HIF transcriptional activity. Specifically, under normal oxygen conditions, α-subunits are hydroxylated in two conserved proline residues (405 and 531) within the O_2_-dependent degradation domain, by prolyl-hydroxylases domain proteins (PHDs; Epstein et al., 2001). The hydroxylation of α-subunit function as a target for von Hippel-Lindau tumor suppressor protein (pVHL), and is subsequently ubiquitylated by the Elongin BC/Cul2/pVHL ubiquitin–ligase complex, a marker for 26S proteasome degradation (Maxwell et al., 1999; Ivan et al., 2001; Jaakkola et al., 2001; Greer et al., 2012). HIF-α subunit is also hydroxylated in an asparagine residue (803) that blocks the association with coactivators. This hydroxylation is mediated by an asparaginyl hydroxylase factor inhibiting HIF-1 (FIH-1; Lando et al., 2002). Together, the oxygen-dependent enzymes, prolyl and asparaginyl hydroxylases, are critical regulatory components of the hypoxic response pathway, regulating both stability and transcriptional activity of HIF complex (Greer et al., 2012; Figure 2).

The canonical pathway of HIF activation defines that under low O_2_ concentration, α-subunits are no longer hydroxylated by PHDs and neither degraded by the proteasome. Instead, this promotes stabilization of α-subunit that translocates to the nucleus and dimerizes with ARNT/HIF-1β subunit recruiting coactivators such as Creb Binding Protein (CBP)/p300. This transcriptional complex binds to hypoxia-response elements (HREs), within the promoter of target genes, and transactivates gene expression that regulates the adaptive response to hypoxia (Semenza et al., 1991; Greer et al., 2012; Semenza, 2014; Figure 2).

In addition to hypoxia, HIF stabilization and transcription activity can be regulated by several other factors, such as reactive oxygen species (ROS), nitric oxide (NO), metabolic intermediates of the tricarboxylic acid (TCA) cycle (such as succinate and fumarate), proinflammatory mediators [tumor necrosis factor-α (TNF-α), interleukine-1β (IL-1β)] and also hormonal factors. The regulation of HIF can occur by increasing its stabilization, by inhibiting both enzymes PHD and FIH, and also by the recruitment and posttranslational modification of cofactors that potentiate the transcriptional complex (Lando et al., 2002).

Hypoxia-inducible factor regulates several genes that promote the adaptation to hypoxia [for example, vascular endothelial growth factor (VEGF), erythropoietin, and glucose transporter-1 (GLUT1)] and also genes that promote a metabolic switch to glycolysis through the transcriptional regulation of key metabolic enzymes [for example, pyruvate dehydrogenase kinase 1 (PDK1), lactate dehydrogenase A (LDHA), and glycogen phosphorylase L (PYGL)]. The transcriptional regulation of these metabolic enzymes by HIF-1 activation under oxygen deprivation stimulates glucose intake, glycolysis, and lactate production to provide ATP. In liver, hypoxia-activated HIF-1 leads to an increase hepatic glucose production by transactivation of phosphoenolpyruvate carboxykinase (PEPCK), a rate-limiting enzyme in gluconeogenesis, gene in liver (Choi et al., 2005).

In obesity, the expansion of adipose tissue (hypertrophy and hyperplasia) creates hypoxia niches that are involved in the inflammatory process and insulin resistance that culminates in hyperglycemia (Halberg et al., 2009; Fujisaka et al., 2013). HIF-1α mRNA and protein are enhanced in adipose tissue in a model of diet-induced obesity, by a mechanism that involves adipogenesis, insulin, and hypoxia (He et al., 2011). Both in obese patients and in animal models of obesity, there is an association between reduced oxygen pressure in adipose depots and macrophage and T cell infiltration (Ye et al., 2007; Pasarica et al., 2009; Fujisaka et al., 2013), highlighting the role of HIF pathway in the development of inflammation.

In the last decade, it has been suggested a broad range of functions including embryonic development, immunity and metabolism (Frede et al., 2006; Semenza, 2014; Corcoran and O’Neill, 2016). When changes in metabolism occur, multiple signals (O_2_, CO_2_, ROS, immunometabolism, and ATP) emanate from the mitochondria. These signals regulate HIF response, which in turn promote major changes in immune cell function and subsequently affects distinct cellular and organic functions (Peyssonnaux et al., 2007; Jantsch et al., 2008; Corcoran and O’Neill, 2016). Therefore, HIF is a key cellular metabolic sensor all over the body.

### Immune Regulation of HIF

Despite the fact that HIF regulation has primarily been thought oxygen concentration, several studies have showed that HIF-1α can have an oxygen-independent regulation, such as in response to hyperglycemia, oxidative stress, and inflammatory proteins (Cramer et al., 2003; Catrina, 2014; Corcoran and O’Neill, 2016). Hypoxia is an important factor in the pathophysiology of a number of human diseases (e.g., obesity, insulin resistance, diabetes, Alzheimer disease, and aging) that are characterized by the presence of inflammatory processes (Eltzschig and Carmeliet, 2011; Iyalomhe et al., 2017). Hypoxia can also lead to the production of oxygen radicals, which, as well, have important implications in these diseases. Nevertheless, HIF-1α can also act as a neuroprotective factor following some neuronal insults, activating survival genes, and promoting the adaptation to oxidative stress and inflammation.

Proinflammatory cytokines (TNF-α and IL-1β) can upregulate HIF-1α, by a mechanism that inhibits PHD enzymes with stabilization of HIF activity; conversely, HIF-1 transcriptional activity can downregulate receptors for inflammatory cytokines, attenuating the neuroinflammation (Dehne and Brune, 2009; Xing and Lu, 2016; Iyalomhe et al., 2017).

The molecular mechanism of HIF activation induced by neuroinflammation involves the activation of TLRs and subsequent activation of the mitogen-activated protein kinase (MAPK) pathway and nuclear factor kappa-light-chain-enhancer of activated B cells (NF-kb), which transactivate HIF-1α under normal oxygen tension (Frede et al., 2006; Spirig et al., 2010). Neuroinflammation induced upregulation of HIF-1α suggests the involvement of neuronal survival pathway during inflammatory neurological insults, particularly in the early stages of the process.

In hypothalamus, HIF-1α is increased after short-term high-fat diet consumption, before the onset of body weight gain (Gaspar et al., 2018). The inhibition of HIF signaling in ARC of mice fed a HFD induces hypothalamic inflammation, with increased expression of TLR4, TNFα, and IL-1β, and is associated with an increase in body mass gain and glucose intolerance (Gaspar et al., 2018). Taken together, these results and the fact that consumption of saturated fats is involved in the biogenesis of obesity (Thaler et al., 2012; Valdearcos et al., 2014), it is possible that HIF-1α stabilization is a regulator and a protective factor against saturated fatty acids induced-neuroinflammation, regulating energy expenditure and metabolism.

## Central Regulation of Metabolism by HIF

The hypothalamus has a critical role in the regulation of energy homeostasis and whole-body metabolism. Changes in hypothalamic nutrient and hormonal sensors play important roles in the genesis of obesity and metabolic disorders. Studies performed at high altitude, in conditions of hypobaric hypoxia, have demonstrated a progressive loss of body mass, caused by the decrease in food intake and thus inducing an energy deficit possibly due to hypobaric hypoxia (Westerterp-Plantenga et al., 1999; Westerterp and Kayser, 2006). In these studies, Wasterterp and colleagues exposed eight men for 31 days to simulated stay in high altitudes with palatable food provided *ad libitum*. They observed a decrease in body mass in 5 ± 2 kg with a 40% reduction in energy intake as a result of a decrease in appetite. Changes in appetite can occur by central mechanisms of energy homeostasis regulation, but also by an increase in leptin secretion (Tschop et al., 1998). Weight loss due to hypobaric hypoxia was also observed in obese patients; in high altitude, obese subjects presented increased leptin levels and higher metabolic rate (Lippl et al., 2010). Populations that live at high altitude had a lower prevalence of impaired fasting glucose and type 2 diabetes compared with low altitude living populations (Santos et al., 2001).

The combination of these studies and the fact that hypothalamus, mainly the ARC, express high levels of both HIF-1α and HIF-2α (Zhang et al., 2011; Gaspar et al., 2018), raised the question of how central HIF activity regulates food intake and whole-body metabolism. HIF-1α is expressed in POMC neurons, in astrocytes and microglial cells within the ARC (Gaspar et al., 2018).

Mice with a neuronal-specific loss for the FIH-1α presented reduced body weight, decreased fat accumulation, elevated metabolic rate, hyperventilation, enhanced insulin sensitivity, and improved glucose and lipid metabolism; in addition, they are resistant to body weight gain and hepatic steatosis induced by high-fat feeding (Zhang et al., 2010). However, this study does not demonstrate which brain region was responsible for this metabolic regulation, since FIH was deleted in whole-body neuronal cells. FIH neuronal deletion, and consequently increased activity of HIF-1a induces an increased in food intake besides the beneficial effects of whole body weight. FIH is an enzyme that is negative regulator of other proteins such SOCS (Ferguson et al., 2007), that suppress leptin signaling. FIH can also regulate several other proteins such as Notch receptors, p105 and IkBa (Cockman et al., 2006; Coleman et al., 2007), which make a difficult task to for the determination of direct FIH action as a regulator of food intake trough HIF signaling. Since many of these metabolic processes are controlled by neuronal regulatory circuits located in the hypothalamus, at least in part the neuronal HIF-1α activity regulates systemic metabolism through the modulation of afferent neural connections.

Another study showed that HIF is present in the hypothalamus and is upregulated by the local availability of glucose and its metabolites (Zhang et al., 2011). In this study, the authors demonstrate that under glucose availability in HIF can function as a glucose sensor that specifically binds to the promoter of pomc gene, upregulation the POMC expression. HIF loss-of-function specifically in POMC neurons caused increase in food intake, decrease in basal metabolism, and consequently increasing fat mass and weight gain to promote obesity development (Zhang et al., 2011).

**FIGURE 3:**
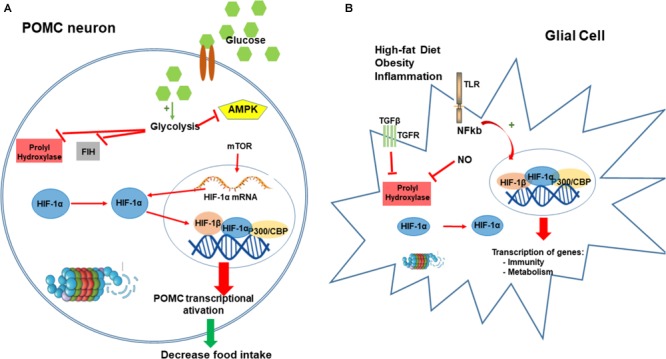
Hypoxia-inducible factor-1 regulation of food intake. HIF in POMC neurons can function as a glucose sensor. After a meal, the increase in neuronal concentration activates neuronal glycolysis producing pyruvate, lactate, and intermediaries from the tricarboxylic acid (TCA) cycle. These metabolites inhibit PHD and FIH enzymes leading to the HIF stabilization. Conversely, these metabolites also block AMPK activation that regulates and increase the expression of HIF-α mRNA. Increase in HIF induces its translocation to the nucleus to bind POMC promoter and consequently stimulating the transactivation of POMC **(A)**. Obesity and consumption of high-fat diet induces an inflammatory condition in the hypothalamus, mostly driven by microglia and glial cells. Our main hypothesis is that inflammation in hypothalamic glial cells (increased TGF-β, TLR4 signaling, and increased NO production) leads to a stabilization of HIF that consequently transactivates several genes involved in the regulation of metabolism and inflammatory process as a compensatory mechanism for the nutritional excess **(B)**. CBP/p300, Creb Binding Protein/p300; PHD, prolyl hydroxylases; FIH-1, factor inhibiting HIF-1; AMPK, 5’ AMP-activated protein kinase; POMC, proopiomelanocortin.

Furthermore, endothelial HIF in hypothalamus also has a main role for central regulation of metabolism. The knockdown of endothelium HIF-1α, resulted in impairment of glucose uptake in the ARC and decreased POMC neuronal activity; following caloric deprivation, mice presented increased caloric intake as compared to control (Varela et al., 2017). Taken together, both studies suggested that hypothalamic HIF regulates glucose uptake in the ARC and consequently controls neuronal POMC adaptation to the changing metabolic environment, important for regulation of energy homeostasis mechanisms (Figure 3A).

Recently, we demonstrated using bioinformatics analysis, that hypothalamic HIF-1 transcripts are directly correlated with hypothalamic transcripts for proteins involved in inflammation, apoptosis and autophagy; in addition, hypothalamic HIF-1 expression is directly correlated with the phenotype of increased energy expenditure (Gaspar et al., 2018). High-fat diet induced increases in protein expression of hypothalamic HIF-1α; ARC inhibition of HIF-1 resulted in further increase in body mass, decreased basal metabolic rate, increased hypothalamic inflammation, and glucose intolerance (Gaspar et al., 2018; Figure 3B).

Orexins are hypothalamic neuropeptides involved in the regulatory network of food intake. One of the orexin-responsive genes is HIF-1α; upon orexin stimulus, HIF-1α gene expression is increased, resulting in increased glucose uptake and glycolytic activity. This provides evidence to support the important role of HIF-1 as a transcription factor in the hormone-mediated regulation of hunger (Sikder and Kodadek, 2007).

Also in the liver HIF proteins have important roles in the regulation of glucose and lipid metabolism. Diet-induced obesity induces an upregulation of hepatic HIF-1α (Ochiai et al., 2011), as a compensatory mechanism for metabolic regulation. Mice with liver-specific deletion of HIF-1α exhibited more severe impairment of glucose tolerance and peripheral insulin-resistance than control littermates; these changes were accompanied by a significant reduction of hepatic glucose uptake (Ochiai et al., 2011). Mice with liver specific deletion of HIF-1β, had increased hepatic gluconeogenesis, increased lipogenic gene expression, and impaired glucose tolerance, similar to what happened in type 2 diabetes (Wang et al., 2009).

Despite the importance hypothalamic and hepatic HIF-1α as regulator of energy, glucose, and lipid homeostasis, the increased expression/stability of HIF-1α during the expansion of white adipose tissue is implicated in the inflammatory process and insulin resistance (Ye et al., 2007; Pasarica et al., 2009; Fujisaka et al., 2013; Sun et al., 2013). Both in obese patients and in animal models of obesity, there is an association between reduced oxygen pressure in adipose depots and macrophage and T cell infiltration (Ye et al., 2007; Pasarica et al., 2009; Fujisaka et al., 2013; Lee et al., 2014; Taylor et al., 2014), highlighting the role of HIF pathway in the development of inflammation. However, obese mice deficient in adipocyte-specific HIF2 displayed dysregulation in thermogenesis under cold exposure, as a result of reduced levels of UCP1 and brown adipose tissue impairment (Garcia-Martin et al., 2015). In fact, in brown adipose tissue, HIF complex (isoform 2) is one of the factors that contribute to the upregulation of thermogenesis as an adaptation to obesity (protective effect) through UCP-1 expression (Vucetic et al., 2011; Garcia-Martin et al., 2015).

Thus, HIF-1 can control BAT activity through both direct and indirect mechanisms. In addition, the induction of HIF activity in neuronal cells (neuron-specific FIH null mice) is involved in the regulation of respiration, energy balance, and lipid metabolism (Zhang et al., 2010). Thus, HIF has emerged as an important component of the hypothalamic neuronal machinery involved in the control of body mass and metabolism (Zhang et al., 2011).

In conclusion, HIF has important functions in the regulation of whole body energy homeostasis (both in regulation of food intake, but also in thermogenesis) and also in the hepatic glucose and lipid metabolism. These functions are both directly in the peripheral organs, but also through central nervous system regulation (Zhang et al., 2010, 2011; Gaspar et al., 2016). Considering the positive metabolic results observed in patients under high altitude (hypobaric hypoxia), it is suggested that whole body activation of HIF signaling can have more beneficial than pathological effects against metabolic complications. However, caution should be taken since chronic systemic HIF1α inactivation (genetic or pharmacological manipulation) attenuates WAT expansion and obesity (Sun et al., 2013; Soro-Arnaiz et al., 2016) despite of the HIF1a-dependent beneficial metabolic effects in the hypothalamus. Specifically, in the hypothalamic, HIF-1α is an important nutrient sensor and regulator of energy metabolism; it is also implicated in the regulation of hypothalamic neuroinflammation during the development of obesity. Hypothalamic inhibition of HIF-1α aggravates obesity-associated metabolic phenotype; this highlights the hypothalamic HIF-1α as a potential target for therapeutic intervention against obesity.

## Concluding Remarks

Both human and experimental data support an important role for hypothalamic HIF-1 in the regulation body weight, glucose homeostasis, and liver metabolism. Modulation of specific and selective central/ hypothalamic HIF in obesity can function as a protective mechanism to reduce the negative impact of hypothalamic dysfunction thus, placing tissue specific HIF-1 activation as a potential therapeutic target for the pharmacological management of obesity and type 2 diabetes. Along the role of HIF-1α in the white adipose tissue development, it might be possible that HIF1α actions favoring obesity in WAT overrides that HIF1α-dependent repression of food intake. Nevertheless, further biological, pharmacological, and clinical studies are needed to provide an extended understanding of the mechanisms and molecular players involved in the different cellular responses of HIF in the central mechanisms of energy homeostasis and also during the course of development of obesity and diabetes.

## Author Contributions

Both authors contributed to the writing, editing, and discussion of the manuscript.

## Conflict of Interest Statement

The authors declare that the research was conducted in the absence of any commercial or financial relationships that could be construed as a potential conflict of interest.
